# Investigating Shear Stress of Ice Accumulated on Surfaces with Various Roughnesses: Effects of a Quasi-Water Layer

**DOI:** 10.1021/acs.langmuir.4c00617

**Published:** 2024-07-02

**Authors:** Xinjiao Cui, Chao Yang, Qiangqiang Sun, Wenqiang Zhang, Xinyu Wang

**Affiliations:** † Institute of Thermal Science and Technology, Shandong University, Jinan 250061, China; ‡ Institute for Advanced Technology, Shandong University, Jinan 250061, China; § Faculty of Engineering, University of Nottingham, Nottingham NG7 2RD, U.K.; ∥ School of Mechatronical Engineering, Beijing Institute of Technology, Beijing 100081, China; ⊥ Shenzhen Research Institute of Shandong University, Shenzhen 518057, China

## Abstract

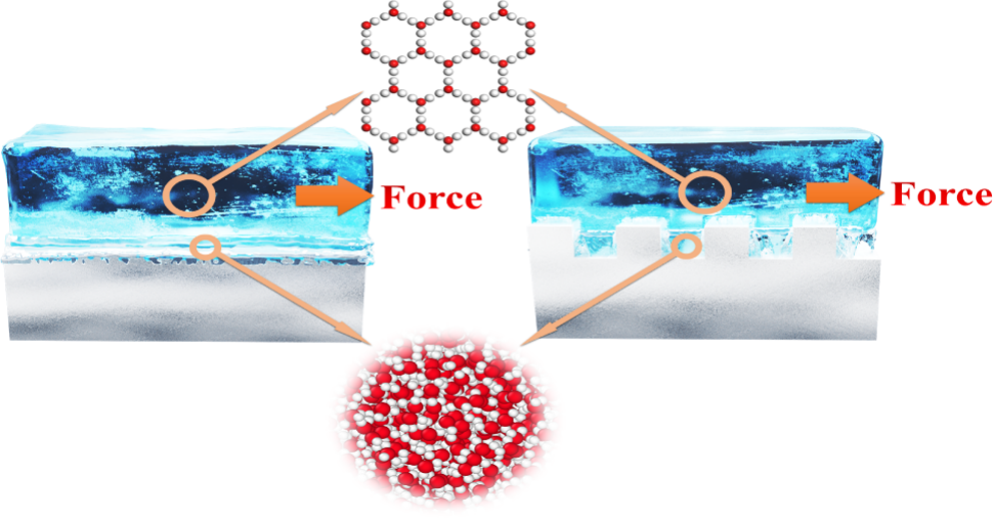

The investigation of the anti-icing/deicing is essential because the icing phenomenon deteriorates the natural environment and various projects. By conducting molecular dynamics simulation, this work analyzes the effect of the quasi-water layer on the ice shear stress over smooth and rough surfaces, along with the underlying physics of the quasi-water layer. The results indicate that the thickness of the quasi-water layer monotonically increases with temperature, resulting in a monotonic decrease in the ice shear stress on the smooth surface. Due to the joint effects of the smooth surface wettability and the quasi-water layer, the ice shear stress increases and then decreases to almost a constant value when the surface changes from a hydrophobic to a hydrophilic one. For rough surfaces with stripe nanostructures, when the width of the bump for one case equals the depression for the other case, the variations of shear stress with height for these two cases are almost the same. The rough surface is effective in reducing the ice shear stress compared to the smooth surface due to the thickening of the quasi-water layer. Each molecule in the quasi-water layer and its four nearest neighboring molecules gradually form a tetrahedral ice-like structure along the direction away from the surface. The radial distribution function also shows that the quasi-water layer resembles the liquid water rather than the ice structure. These findings shed light on developing anti-icing and deicing techniques.

## Introduction

Icing is a significant issue in low-temperature environments, with serious implications for transportation, energy supply, and infrastructure maintenance.^1−3^ Generally, the research attention mainly focuses on the anti-icing and deicing technologies to address this problem. Anti-icing aims to prevent ice formation, while deicing involves removing ice that has already formed. In recent years, researchers have made significant contributions to passive anti-icing, heat melting, chemical melting, and mechanical deicing.^4−7^ However, the existing methods still have some challenges. For example, some of them are ineffective, costly, and difficult to cope with changes in different environmental conditions. Therefore, it is crucial to conduct research on optimizing existing technologies or developing new anti-icing and deicing methods.

Reduction of the ice adhesion stress is the core of the anti-icing and deicing efforts,^8^ due to the fact that ice can be removed by natural forces when the adhesion stress between the ice and wall surface is less than 10 kPa.^9^ Ice adhesion is primarily influenced by numerous factors of the substrate, i.e., material,^10,11^ structure,^12,13^ temperature,^8,10,14^ wettability,^10,15,16^ and elasticity.^17,18^ In addition, in recent years, many researchers have investigated how surface morphology affects ice adhesion, while the effects of surface roughness and doping on ice adhesion are not discussed actively. Ruan et al.^12^ computed ice tensile and shear stress on Al-terminated α-Al_2_O_3_(0001) surfaces with different morphologies and dopant atoms at the nanoscale. The results show that the ice adhesion depends on the solvent-accessible surface area and nonbonding interactions, and doping iron atoms on the surface can reduce the ice adhesion stress. Also, the ice adhesion can be well controlled by adjusting the temperature.^14^ Moreover, although the formation of ice is unavoidable at specific conditions, the adhesion stress of ice on the superhydrophobic surface generally is low, which ensures that it is a promising material in anti-icing/deicing.^15,16,19−21^ A deformable surface is also a solution for anti-icing/deicing^17^ because it usually shows low ice adhesion and satisfying durability.^18^ After examining the effects of surface wettability and elasticity on the ice tensile and shear stress, Ibáñez-Ibáñez et al.^17^ concluded that elasticity has a more significant impact on reducing the ice shear stress compared with hydrophobicity. To reduce the ice adhesion stress, some materials such as lubricants,^6,22,23^ graphene,^24,25^ or carbon nanotubes^24,26^ also can be coated on the substrate surface.

Faraday first proposed the existence of a macroscopic quasi-water/premelting layer on the surface of ice even if the temperature is lower than the melting point in the 19th century.^27,28^ In terms of the nature, structure, thickness, and temperature-dependence of the premelting layer, it has been debated for more than 160 years because the premelting phenomenon of ice affects many phenomena on Earth, i.e., the origin and lifetime of clouds, the retreat of ice floes in the Arctic and Antarctic, the electrification of thunderclouds, and potholes in roads. In general, the onset of premelting depends on the bulk melting temperature. Below the melting point at about 10–20 K, the thickness of the quasi-water layer is around 1 nm,^10,29^ and it becomes thick when the system temperature approaches the melting point.^30^ Due to the difficulty in distinguishing the spectroscopic profile of the quasi-water region from that of liquid water, the quasi-water layer is usually considered to resemble liquid water.^31^ The quasi-water layer formed spontaneously under pressure-induced or friction-heating conditions exhibits hydrodynamic properties similar to those of the bulk supercooled water.^32^ In addition, van der Waals forces also play a role at the ice–water interface, and Luengo–Márquez et al.^33^ quantitatively modeled the surface free energy of the premelting layer by computing structural forces.

The presence of a quasi-water layer has been considered as a convincing explanation for the low adhesion stress of ice.^10,34,35^ Baran et al.^32^ showed that the frictional behavior of ice on the atomic scale is influenced by spontaneous premelting, pressure melting, and frictional heating. Also, they believed that a quasi-water layer could be described quantitatively by a model of bulk Couette flow with slip. Lin et al.^11^ showed that the adhesion stress of ice on a solid substrate is mainly determined by the quasi-water layer through molecular dynamics (MD) simulations. Emelyanenko et al.^8^ discussed the temperature-dependent quasi-water layer and its influences on ice adhesion, focusing on superhydrophobic surfaces.

Despite the numerous studies conducted in recent years, the predictions of premelting temperatures and quasi-water layer thicknesses remain ambiguous. For example, previous experimental efforts were unavoidably affected by a certain amount of uncertainties, such as the thickness of the quasi-water layer dramatically affected by the measurement methods and impurities.^10,30^ Moreover, the impacts of the quasi-water layer on the ice shear stress of various surfaces are not discussed actively, and the influence of roughness on the quasi-water layer has not been systematically studied. Therefore, in this study, we focus on elucidating the physics of the quasi-water layer and its influences on the shear stress of ice on smooth/rough surfaces with various temperatures and wettabilities. The rest of the paper is organized as follows: the simulation details, the measuring method of the ice shear stress, and the criteria distinguishing the quasi-water layer from bulk ice molecules are presented in the Methodology section. The effects of the quasi-water layer on the shear stress of ice on the smooth or rough surfaces are reported in the Effects of Temperature and Wettability on Ice Shear Stress on the Smooth Surfaces section and Effect of Surface Roughness on Ice Shear Stress section, respectively. Finally, the main conclusions are summarized in the Conclusions section.

## Methodology

**Figure 1:**
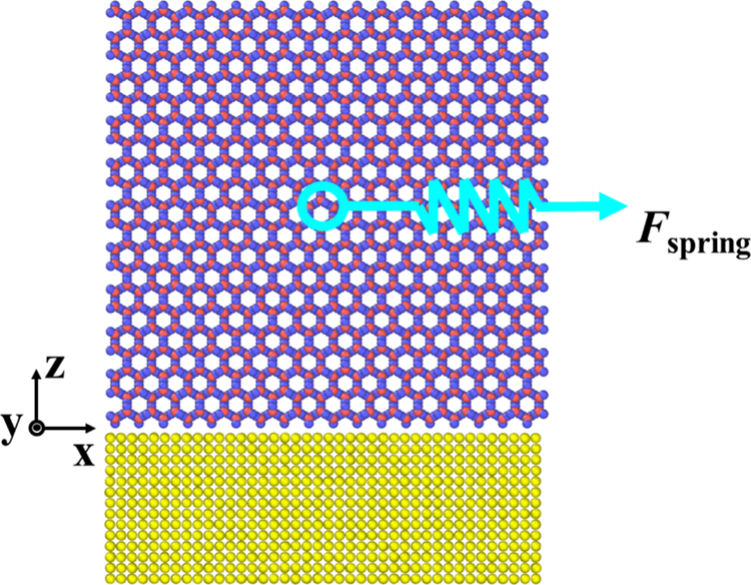
MD simulation system of ice and Ag. The red, blue, and yellow particles are oxygen, hydrogen, and Ag atoms, respectively.

To reveal the interfacial adhesion properties of hexagonal ice (*I*_h_) on a solid surface at the molecular level, geometrical models of silver and ice crystals are generated. The simulation system for ice and wall is shown in Figure 1, with a dimension of 81 × 44 × 265 Å^3^. The thicknesses of the Ag wall and ice in the *z* direction are 26 and 77 Å, respectively. 6160 wall atoms and 8640 ice molecules are simulated. Periodic boundary conditions are applied in the *x*, *y*, and *z* directions, and a vacuum region is introduced above the ice molecules to eliminate the effect of the periodic boundary conditions on the simulation results.^24^

At the bottom of the silver wall, atoms are constrained to their initial positions via applying virtual springs, and the other silver atoms are free to move. The interaction forces between silver and silver, and silver and ice are calculated from the 12-6 Lennard-Jones (LJ) potential:^10^
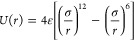
1where ε is the well-depth and σ is the distance where the interparticle potential is zero. The corresponding force field parameters are shown in Table 1.

**Table 1:** Force Field Parameters of the LJ Potential

materials	ε (eV)	σ (Å)
Ag–Ag^36^	0.4080	2.551
Ag–O^36^	0.0611	2.8589
O–O^36^	0.0091	3.1668

In this work, all simulations are performed by the Large-scale Atomic/Molecular Massively Parallel Simulator (LAMMPS) package.^37^ The TIP4P/ice model^38^ where the predicted melting temperature is 269.8 K^39^ is used because it can accurately predict the near-freezing and bulk properties of ice and water.^10^ The hydrogen and oxygen atoms in the ice molecule have charges of 0.5897*e* and −1.1794*e*, respectively.^36^ Also, the bonds and angles of the ice/quasi-water molecules are constrained by the SHAKE algorithm,^40^ and the PPPM method is used to solve the long-range electrostatic interactions. The time step is 1.0 fs, and the temperature of the simulation system is controlled to the desired level by deploying the Langevin thermostat method.^41^ During the simulation, the whole system is first relaxed under the NVT and NVE ensembles for 0.5 and 2 ns, respectively. Then, the entire ice is pulled horizontally using the steered molecular dynamics (SMD) method.

### Steered Molecular Dynamics Method

The steered molecular dynamics^42^ is a nonequilibrium simulation process, where a molecular dynamics simulation is performed by artificially applying an external force to one/many atoms or fixing their positions to control the behavior of the whole system. In the SMD method, a virtual point and a virtual spring^10^ are artificially added, and the center of mass (COM) of the ice is driven to move by the force exerted by the point moving at a constant speed,^43^ as shown in Figure 1. The spring force can be computed from
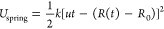
2
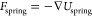
3where *U*_spring_ and *F*_spring_ are the potential energy and the spring force, respectively. *k* is the stiffness coefficient of the virtual spring, and *u* is the moving speed of the virtual point. *k* and *u* are chosen as 41838.8 kJ/(mol^–1^ nm^–2^) and 0.5 nm ns^–1^, respectively. *R*(*t*) and *R*_0_ are the positions of the center of mass of the pulled ice/water at the moment *t* and the initial moment, respectively.

The shear stress of ice can be calculated from the following equation:
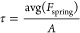
4where τ is the shear stress of the ice, avg(*F*_spring_) is the average force during the pulling of the ice, and *A* is the projected area of the interface between the ice and the wall. In this work, the result for each case reported in the following sections is the average value of three independent simulations.

### Identification of Quasi-Water and Ice Molecules

Distinguishing ice/water is an essential issue in anti-icing and deicing. Various methods were proposed to address the issue, for example, the average number of hydrogen bonds,^36,41,44,45^ tetrahedral order parameter,^36,46,47^ local density profile,^47,48^ Dellago’s Q6 parameter,^49^ and CHILL+ algorithm.^50,51^ A commonly used method is to calculate the average number of hydrogen bonds.^44^ For a perfect ice crystal, each molecule has four neighboring molecules, resulting in the average number of hydrogen bonds being around 3.99.^41^ However, the average number of hydrogen bonds in water is less than 3.99. The geometric distance-angle identification method^36^ is used to identify the hydrogen bonds. In this method, the hydrogen bond exists when the angle (θ) between the OH hydrogen bond and the OH covalent bond and the distance (*r*) between the hydrogen and oxygen atoms on different ice/quasi-water molecules (see Figure S1 for details) satisfy the following conditions:
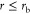
5
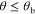
6where *r*_b_ and θ_b_ are the cut-off distance and cut-off angle. Based on previous studies,^45^ the values of these two parameters are chosen as 3.5 Å and 40°, respectively.

The tetrahedral order parameter proposed by Errington et al.^46^ can also effectively distinguish between ice and water, with the parameter calculated as
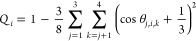
7where *i*, *j*, and *k* are indices of O atoms. The angle θ_*j*,*i*,*k*_ is composed of the O atoms of *j*, *i*, and *k* (the O atom of *i* is the vertex of the angle). The value of *Q_i_* of an ice molecule is 0.95, which agrees well with the value reported by Sánchez et al.^47^

The frequency of the tetrahedral order parameter can be calculated by the following equation:
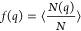
8where *N*(*q*) is the number of ice/quasi-water molecules in the range [*q*, *q* + Δ*q*], *N* is the total number of molecules in the system, and parentheses denote the ensemble average.

In this work, the average number of hydrogen bonds and the tetrahedral order parameter are calculated by the tool command language (TCL) scripts of the visual molecular dynamics (VMD) software.^36^ When a molecule has the average number of hydrogen bonds *N*_h_ < 3.99 or the tetrahedral order parameter *Q* < 0.95, the molecule is identified as a quasi-water molecule. The local density distribution^47,48^ of quasi-water/ice molecules along the *z* direction also can be used to distinguish water from ice.

## Results and Discussion

### Effects of Temperature and Wettability on Ice Shear Stress on the Smooth Surfaces

**Figure 2:**
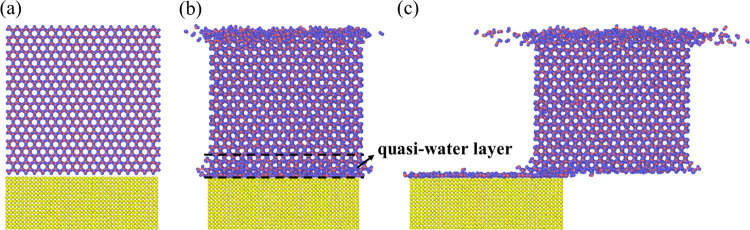
Snapshots of the ice and smooth surface system at 260 K (a) at the initial state, (b) at 2.5 ns when relaxation is completed, and (c) at a time step when ice is being pulled.

In this section, we focus on illustrating the effects of the quasi-water layer thickness on the shear stress of ice accumulated on the smooth surfaces at various temperatures and wettabilities. The sketch of the simulation system is shown in Figure 2, and the SMD method introduced in the Steered Molecular Dynamics Method section 2.1 is utilized to compute the ice shear stress. After relaxation for 2.5 ns, the state of the ice-Ag system changes from the initial moment shown in Figure 2a to that shown in Figure 2b. We can see that the morphology of molecules interacting with the surface or the vacuum region shifts from ice to water. The structure of the outmost molecules interacting with the vacuum domain is disrupted. This is because they are in a free state and move inward to form additional hydrogen bonds, resulting in additional strain and disruption in the initial network of ice hydrogen bonds.^52,53^ The near-surface molecules whose morphologies resemble water are called the quasi-water layer, and the thickness of the layer is affected by temperature and wettability.^30,34^ The pulling process of the ice is presented in Figure 2c, and the ice has slipped some distance relative to the surface while a section of the quasi-water layer remains.

**Figure 3:**
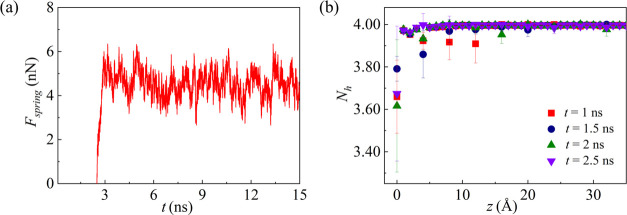
(a) Distribution of time-dependent spring force when ice is detached from the surface. (b) Profile of the average number of hydrogen bonds along the *z* direction.

During the pulling of the ice, the variation of the pulling force is shown in Figure 3a. The pulling force increases gradually from 0 before reaching a stationary state. The average value of the pulling force at the stationary state is defined as the shearing force of the ice, and then the shear stress is computed from eq 4, i.e., the shear stress is 128 MPa at 260 K. As shown in Figure 3b, the average number of hydrogen bonds, *N*_h_, gradually increases along the *z* direction until it reaches about 3.99. Based on the distinguishing criteria of the quasi-water molecules clarified in the Identification of Quasi-Water and Ice Molecules section 2.2, the thickness of the quasi-water layer is about 10 Å at 260 K.

**Figure 4:**
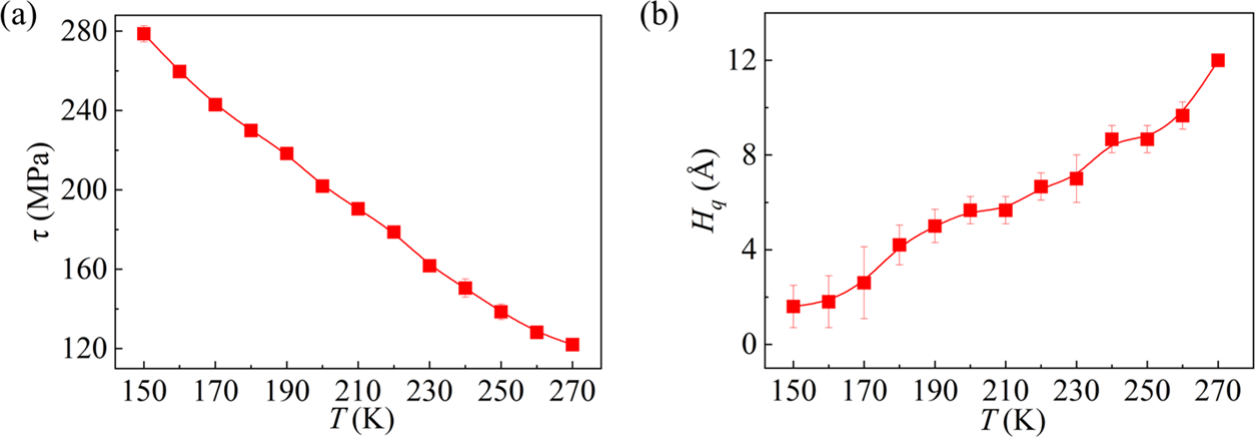
Effect of temperature on (a) ice shear stress and (b) quasi-water layer thickness. Points and curves represent simulation and data fitting results, respectively.

To clarify the effect of temperature on the quasi-water layer thickness and the ice shear stress, simulations are conducted by varying only the temperature of the ice-Ag system, and the corresponding results are shown in Figure 4. In Figure 4a, one can observe that the shear stress of the ice drops almost linearly with increasing of temperature, which is similar to previous experimental findings.^8,54^ Effect of temperature on *H*_q_ is shown in Figure 4b, which is calculated before the ice crystal is pulled. According to reference,^32^ the quasi-water layer tends to reduce the shear stress of ice. Therefore, the reduction of ice shear stress observed in Figure 4a is mainly caused by a thicker quasi-water layer at a higher temperature shown in Figure 4b, which was also observed in experiments conducted by Liljeblad et al.^55^ and Emelyanenko et al.^8^

**Figure 5:**
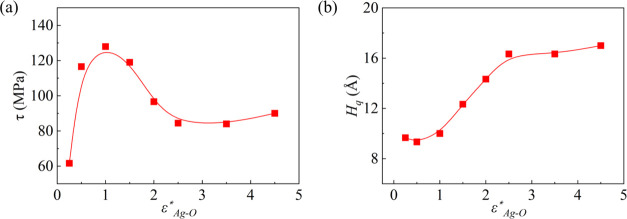
Effect of surface wettability on (a) ice shear stress and (b) quasi-water layer thickness. Points and curves are simulation and data fitting results, respectively.

Then, dimensionless ε*_Ag–O_ = ε_Ag–O_/0.0611 is varied artificially to study its influences on the quasi-water layer thickness and ice shear stress, where 0.0611 is calculated from the Lorentz-Berthelot rule^36,56^ in Table 1. For icing on high-voltage power lines, the typical temperature will be 10–30 K below the freezing point (∼273 K), so we choose the temperature *T* = 260 K for the current simulation. In Figure 5a, the ice shear stress peaks almost at ε*_Ag–O_ = 1 and decreases when the surface becomes more hydrophilic or more hydrophobic represented by larger or smaller ε*_Ag–O_. Figure 5b shows that the thickness of the quasi-water layer gradually increases as the surface becomes hydrophilic. This can be understood from the enhancement in intermolecular interactions between the surface and water. As ε*_Ag–O_ rises, the influence of the surface on nearby molecules becomes significant, resulting in wider disruption of the nearby ice structure.^48^ The larger value of ε*_Ag–O_ featuring a thicker quasi-water layer represents the greater interaction force between the surface and the ice. A thicker quasi-water layer favors the reduction of ice shear stress, while a greater interaction force increases the ice shear stress. Therefore, the variation of ice shear stress observed in Figure 5a is a joint effect of ε*_Ag–O_ and the quasi-water layer thickness.

**Figure 6:**
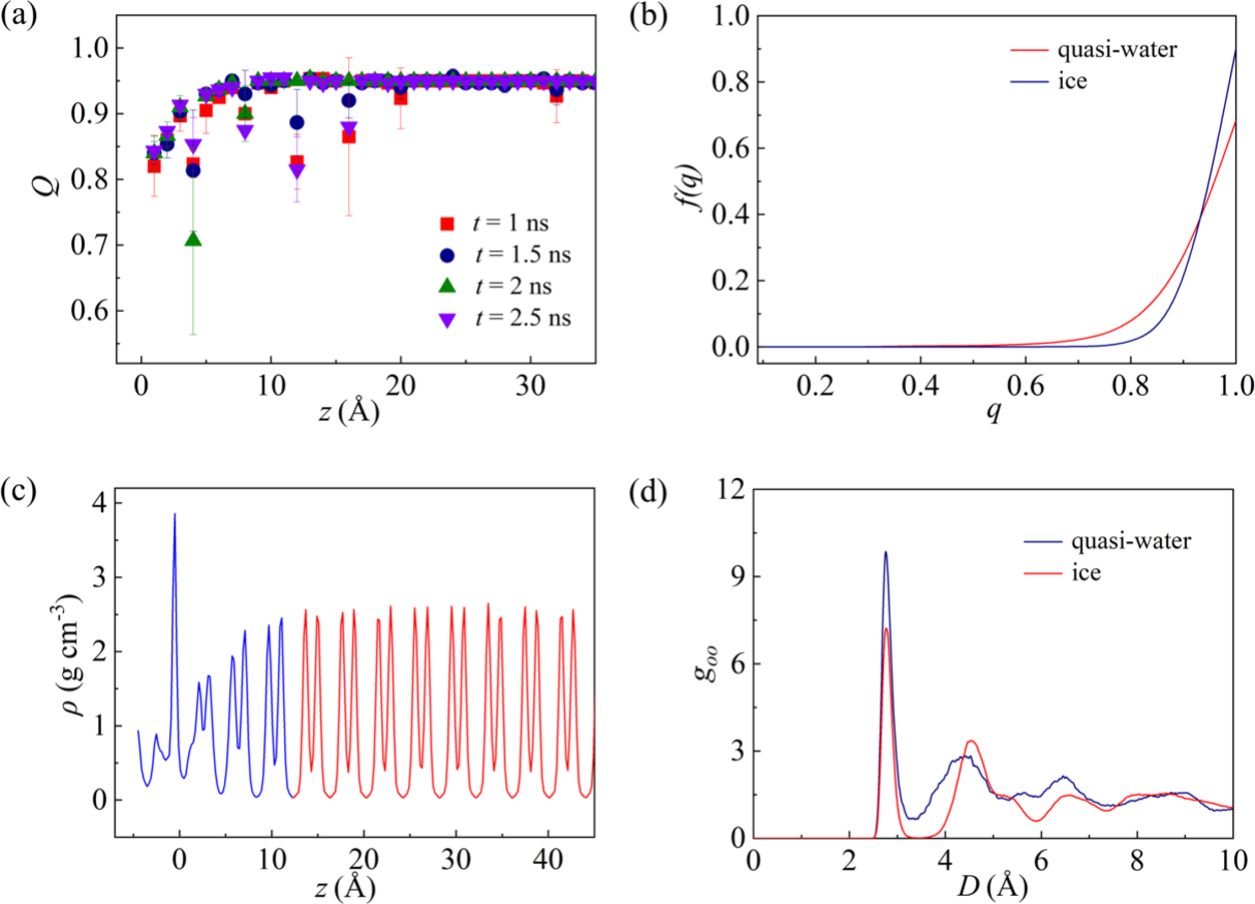
(a) Distribution of the tetrahedral order parameter along the *z* direction, (b) frequency of tetrahedral order parameter, (c) local density profile along the *z* direction, and (d) radial distribution function of the ice-Ag system at 260 K and ε*_Ag–O_ = 1.

The quasi-water layer can be identified not only by the average number of hydrogen bonds in Figure 3b but also by the tetrahedral order parameter. Taking the ice-Ag system at 260 K and ε*_Ag–O_ = 1 as an example, *Q* gradually increases along the *z* direction before reaching almost a constant value of 0.95, as shown in Figure 6a. The tetrahedral order parameter method also yields a quasi-water layer thickness of around 10 Å, which is in good agreement with the value obtained using the average hydrogen bonds method. The structures of the ice and quasi-water layer are further analyzed by examining the frequency of the tetrahedral order parameter, as shown in Figure 6b. One can observe that the tetrahedral order parameter of ice is mainly concentrated around 0.95, while the proportion of the quasi-water layer at around 0.95 is lower compared to ice. This suggests that the quasi-water layer is less structured than ice. Based on the fact that there is a difference in the density distribution between the quasi-water layer and ice and the distance between the local density peaks and valleys decreases at the demarcation,^47^ the quasi-water layer thickness is determined around 10 Å. The quasi-water layer thickness (∼10 Å) determined by the three approaches coincides with each other, validating the reliability of the data (see Figure S2). The radial distribution function (RDF) of the quasi-water region shown in Figure 6d is similar to that of water reported in reference,^47^ representing that the quasi-water region is similar to liquid water. Liljeblad et al.^55^ likewise came to this conclusion after observing that the vibrational spectra of the quasi-water region are clearly different from those of ice. So, the three criteria are equivalent in demonstrating the thickness of the quasi-water layer resembling liquid water.

### Effect of Surface Roughness on Ice Shear Stress

**Figure 7:**
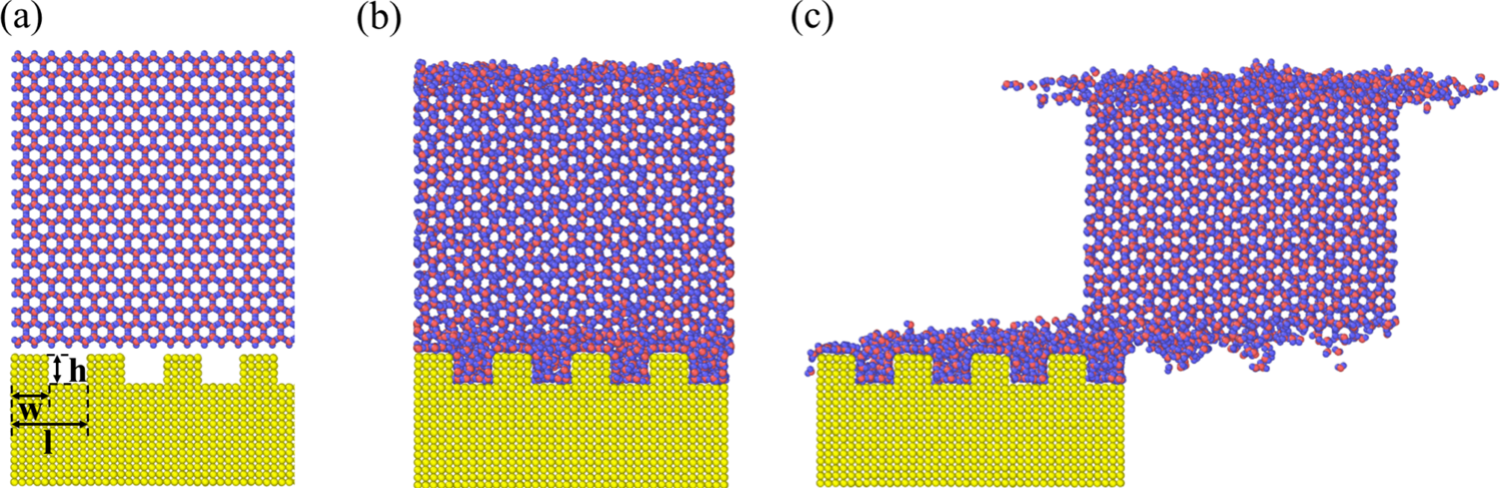
Snapshots of the ice and rough surface system at 260 K (a) at the initial state, (b) at 2.5 ns when relaxation is completed, and (c) at a time step when ice is being pulled.

To clarify the impact of the surface roughness on quasi-water layer thickness and ice shear stress, the surface structure is modified. The width (*w*), height (*h*), and cycle length (*l*) of the rough surface bumps are defined in Figure 7a, with the bumps arranged as stripe nanostructures along the *y* direction. After relaxation for 2.5 ns, the initial structure shown in Figure 7a becomes the one in Figure 7b. The interfacial quasi-water layer remains visible, with quasi-water molecules penetrating into the depressions of the rough surface. During the pulling process of the ice, the quasi-water layer can also be observed, as shown in Figure 7c.

**Figure 8:**
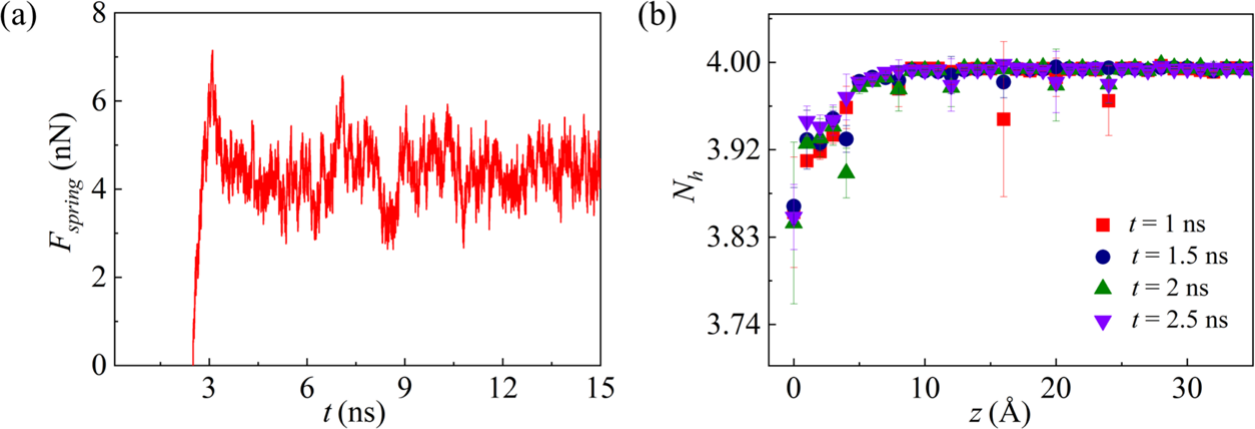
(a) Profile of the time-dependent spring force and (b) the average number of hydrogen bonds along the *z* direction at *w* = 10 Å and *h* = 8 Å.

Figure 8 shows the spring force and the average number of hydrogen bonds at *w* = 10 Å and *h* = 8 Å. The ice shear stress and the quasi-water layer thickness are 123 MPa and 11 Å, respectively.

**Figure 9:**
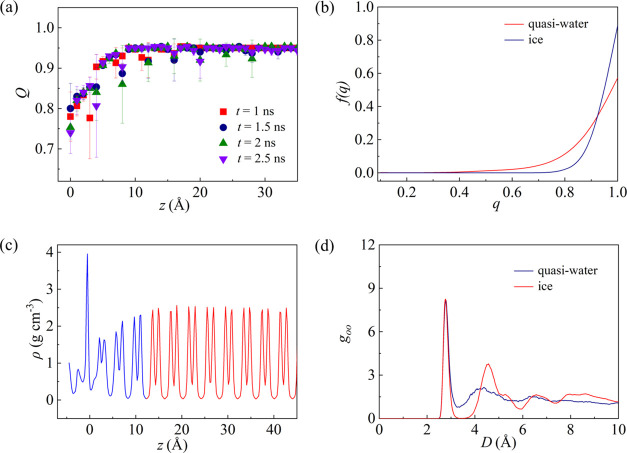
(a) Distribution of the tetrahedral order parameter along the *z* direction, (b) frequency of tetrahedral order parameter, (c) local density profile along the *z* direction, and (d) radial distribution function of the ice-Ag system at *w* = 10 Å and *h* = 8 Å.

Similar to the smooth surface, the thickness of the quasi-water layer on the rough surface can be also determined from Figure 9a,c, and these three methods give the same quasi-water layer thickness. The frequency of the tetrahedral order parameter shown in Figure 9b is consistent with the results for the smooth surface in Figure 6b, indicating that the structure of the quasi-water layer on the rough surface resembles that on the smooth surface. Moreover, the quasi-water layer on the rough surface exhibits similarities to liquid water, as can be seen from the RDF shown in Figure 9d.

**Figure 10:**
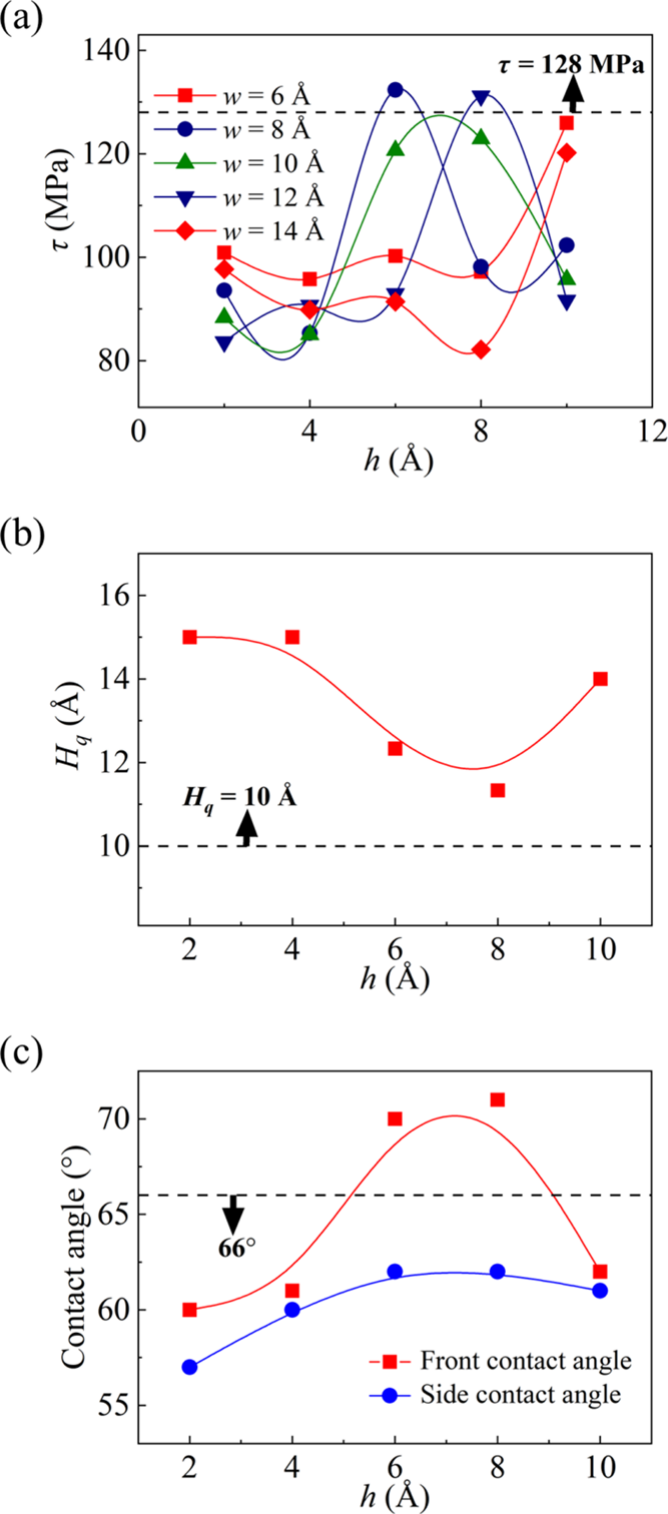
(a) Ice shear stresses on the surfaces with various rough elements, (b) thickness of the quasi-water layer, (c) contact angle at different heights at *w* = 10 Å.

The effects of surface roughness on the quasi-water layer thickness as well as the ice shear stress are investigated by varying the width and height of the surface bumps while maintaining other parameters as constant values (i.e., *l* = 20 Å, *T* = 260 K and ε*_Ag–O_ = 1). All cases are summarized in Table 2. The ice shear stresses at different widths and heights are shown in Figure 10a. At *w* = 6 and 14 Å, the ice shear stress remains almost a constant value before showing an upward trend. Moreover, an increase followed by a decrease is observed for *w* = 8 or 12 Å. It is worth noting that the sum of the bump widths for these two cases is equal to the cycle length of 20 Å, that is, the width of the bump for one case equals that of the depression for the other case, where the trend of ice shear stress with height is similar for both cases.

**Table 2:** Characteristics of Studied Surfaces

number	*w*	*h*	*l*
1	6	2	20
2	6	4	20
3	6	6	20
4	6	8	20
5	6	10	20
6	8	2	20
7	8	4	20
8	8	6	20
9	8	8	20
10	8	10	20
11	10	2	20
12	10	4	20
13	10	6	20
14	10	8	20
15	10	10	20
16	12	2	20
17	12	4	20
18	12	6	20
19	12	8	20
20	12	10	20
21	14	2	20
22	14	4	20
23	14	6	20
24	14	8	20
25	14	10	20

Using *w* = 10 Å as an example, the effects of the height of the rough elements on the ice shear stress and the quasi-water layer thickness are illustrated in Figure 10a,b, respectively. When the height of the rough elements grows, the ice-wall contact area expands, which tends to enlarge the ice shear stress.^57^ Thus, it can be seen that the ice shear stress decreases due to the thickening of the quasi-water layer when *h* gradually grows (*h* ≥ 7 Å). When *h* gets progressively shorter (*h* < 7 Å), however, the reduction of the ice shear stress is a combined effect of the thickening quasi-water layer and small ice-wall contact area. The quasi-water layer thickness is affected by the surface roughness, which impacts the surface wettability. As shown in Figure 10c, the front and side contact angles of liquid water at different heights are measured (see SI section 3 for details). According to the relationship between contact angle and wettability,^58,59^ a smaller contact angle represents a more hydrophilic surface, which features a thicker quasi-water layer and consequently lower ice shear stress. Conversely, a larger contact angle indicates a more hydrophobic surface, leading to a thinner quasi-water layer and higher ice shear stress. This is in good agreement with the previous work where the small contact angle of the rough surface favors the reduction of the ice shear stress.^60^ This phenomenon is consistent with the findings in Figure 5b. However, the surface wettability is artificially varied by adjusting the interaction force or roughness in the previous and this sections, respectively, which is responsible for the difference between Figures 5 and 10.

The ice shear stress is 128 MPa and the quasi-water layer thickness is 10 Å on the smooth surface mentioned in the Effects of Temperature and Wettability on Ice Shear Stress on the Smooth Surfaces section 3.1. The rough surfaces facilitate the reduction of ice shear stress through an increase in the thickness of the quasi-water layer, as shown in Figure 10a,b. This is mainly due to that the droplets tend to spread along the *y* direction of the stripe nanostructures, resulting in lower side contact angle than the smooth surface contact angle of 66° shown in Figure 10c. Therefore, we can adopt a stripe rough surface appropriately to minimize ice shear stress.

## Conclusions

The effects of the quasi-water layer on the ice shear stress on smooth and rough surfaces are investigated using molecular dynamics simulations. The ice shear stress depends on the system temperature, surface wettability, and roughness, and also the quasi-water layer thickness. For the smooth surface, the surface hydrophilicity leads to a thicker quasi-water layer, while the interaction force between the ice and the surface also increases, resulting in a peak in the shear stress. Moreover, the rough surface with stripe nanostructures changes the quasi-water layer thickness by varying the surface wettability. It is also observed that when the width of the bump for one case equals the depression for the other case, the variations of shear stress with height for these two cases are almost the same. Compared to a smooth surface, a further reduction of the ice shear stress is observed for the rough surface, which is caused by the thicker quasi-water layer. Higher temperatures result in lower ice shear stress due to the gradual increase of the thickness of the quasi-water layer. By showing the average number of hydrogen bonds and the tetrahedral order parameter along the surface-normal, each water molecule gradually forms a regular tetrahedral arrangement structure. In addition, the radial distribution function confirms that the molecules in the quasi-water region exhibit characteristics closer to liquid water. To effectively remove ice from the surface with minimal damage, it is essential to minimize the shear stress of the ice. This can be achieved by increasing the wall temperature and quasi-water layer’s thickness, and selecting appropriate wall wettability and roughness. In future work, we will investigate whether fracture of the ice slab occurs at sufficiently low temperatures, by applying a pull only on the upper half of the ice atoms.^55^ Meanwhile, the shear failure stress will be studied as it reflects the maximum force needed to cause the shear movement of the ice layer.

## Data Availability

No data was used for the research described in the article.

## Supporting Information Available

Details of the definition of the hydrogen bond, the quasi-water layer thickness determined by the three approaches and contact angle measurement on various rough surfaces (PDF)

## Nomenclature

*U*(*r*): potential energy
*r*: distance
*U*_spring_: potential energy of a fictitious spring
*F*_spring_: spring force
*k*: stiffness factor of a fictitious spring
*u*: velocity
*t*: time
*R*(*t*): position of the center of mass of ice/water at time t
*R*_0_: position of the center of mass of ice/water at the initial moment
*A*: area of contact between ice and surface
*r*_b_: cut-off distance
*Q*: tetrahedral order parameter
*f*(*q*): frequency of the tetrahedral order parameter
*N*(*q*): number of molecules with tetrahedral order parameters in the range [*q*, *q* + Δ*q*]
*N*: number of atoms
*N*_h_: average number of hydrogen bonds
*H*_q_: thickness of quasi-water layer
*g*_oo_: radial distribution function
*T*: temperature

## Greek Symbols

ε: well-depth
σ: the distance where the interparticle potential is zero
τ: shear adhesion stress
θ: angle
θ_b_: cut-off angle
ρ: density

## Subscripts

*b*: referential value
*h*: hexagonal ice
*i*, *j*, *k*: atom index

## Superscript

*: non-dimensional value
