# Supplementary material for: Investigating Shear Stress of Ice Accumulated on Surfaces with Various Roughnesses: Effects of a Quasi-Water Layer
Source: Langmuir. 2024 Jul 2;40(28):14214–23. doi: 10.1021/acs.langmuir.4c00617 (PMC11256739; doi:10.1021/acs.langmuir.4c00617)
Supplement: Supplementary file 1 — la4c00617_si_001.pdf [file la4c00617_si_001.pdf]

Supporting Information

Investigating Shear Stress of Ice Accumulated on Surfaces with Various Roughnesses: Effects of a Quasi-Water Layer

Xinjiao Cui[a,b], Chao Yang[a], Qiangqiang Sun[c,*], Wenqiang Zhang[d,*], Xinyu Wang[a,e,*]

[a] *Institute of Thermal Science and Technology, Shandong University, Jinan 250061, China*

[b] *Institute for Advanced Technology, Shandong University, Jinan 250061, China*

[c] *Faculty of Engineering, University of Nottingham, Nottingham NG7 2RD, UK*

[d] *School of Mechatronical Engineering, Beijing Institute of Technology, Beijing 100081, China*

[e] *Shenzhen Research Institute of Shandong University, Shenzhen 518057, China*

Number of pages: 3

Number of figures: 3

Number of schemes: 0

Number of tables: 0

[*] *Corresponding authors. E-mail addresses:* sh1225128@sina.com (Q. Sun), wenqiangzhang@bit.edu.cn (W. Zhang), xyw@sdu.edu.cn (X. Wang).

1. Details of the definition of the hydrogen bond

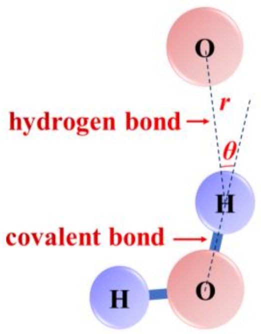

Fig. S1 The definition of the hydrogen bond.

2. The quasi-water layer thickness determined by the three approaches.

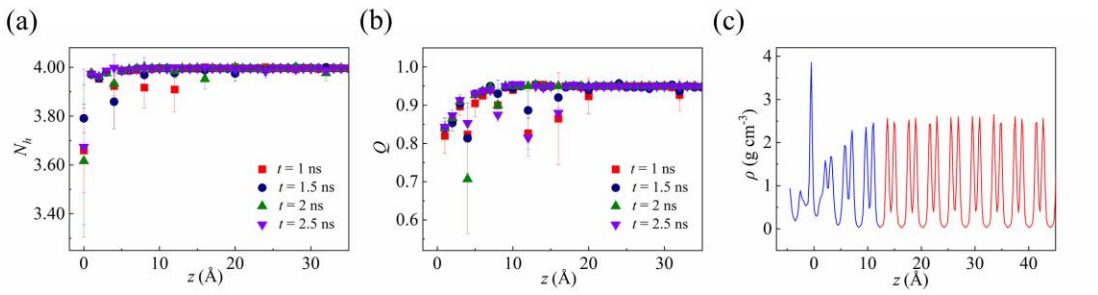

Fig. S2 Profiles of (a) average number of hydrogen bonds, (b) tetrahedral order parameter and (c) local density along the z direction on the smooth surface at 260 K and $\varepsilon^{*}_{\text{Ag-O}} = 1$.

3. Details of contact angle measurement on various rough surfaces

The contact angle of the rough surface is measured. The area of the rough surface is 243 × 264 Å^2, and a water drop with a radius of 60 Å is then placed on it. The configurations of other simulation parameters, such as the temperature control method, potential, and time step, are consistent with those in Section 2. The temperature is maintained at 300 K and the total duration of the simulation is 2 ns. The NVT ensemble is firstly maintained for 0.5 ns, followed by the NVE ensemble for 1.5 ns. The last 1 ns during the NVE ensemble is used to measure the contact angle. Fig. S3 shows the state at the initial and steady moments of

the simulation at w = 10 Å and h = 8 Å, and the front and side contact angles are obtained from Fig. S3b and Fig. S3c respectively.

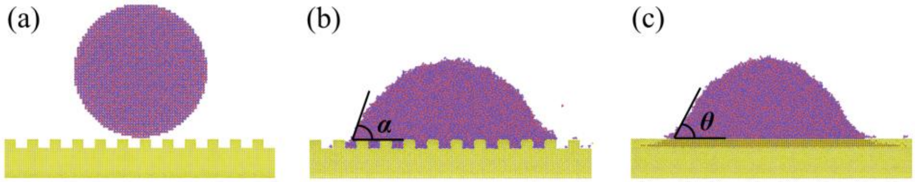

Fig. S3 (a) Initial state, (b) front and (c) side contact angles at the final state of the water drop on the rough surface.
